# The Impact of Resistance Training Program on Static Balance in Multiple Sclerosis Population: A Randomized Controlled Trial Study

**DOI:** 10.3390/jcm11092405

**Published:** 2022-04-25

**Authors:** Luis Andreu-Caravaca, Domingo J. Ramos-Campo, Pedro Manonelles, Linda H. Chung, Salvador Ramallo, Jacobo Á. Rubio-Arias

**Affiliations:** 1 Faculty of Sport, Catholic University of Murcia, 30107 Murcia, Spain; 2 International Chair of Sports Medicine, Catholic University of Murcia, 30107 Murcia, Spain; pmanonelles@ucam.edu; 3 LFE Research Group, Department of Health and Human Performance, Faculty of Physical Activity and Sport Science-INEF, 28001 Madrid, Spain; domingojesus.ramos@upm.es; 4 UCAM Research Center for High Performance, Catholic University of Murcia, 30107 Murcia, Spain; lhchung@ucam.edu; 5 Department of Quantitative Methods for Business and Economics, University of Murcia, 30100 Murcia, Spain; salvador.ramallo@um.es; 6 Health Research Centre, Department of Education, Faculty of Educational Sciences, University of Almería, 04120 Almeria, Spain; jararias@ual.es

**Keywords:** neurological disorders, stabilometry, strength training, postural control

## Abstract

Background: Multiple sclerosis (MS) is a neurological disease that affects balance. Among the non-pharmacological strategies to improve this variable, physical exercise is one of the most widely used. However, the benefits of some types of training, such as resistance training, on static balance in this population are still unclear. This study aims to analyze the effects of a resistance training (RT) intervention on balance in people with MS. Methods: Thirty people with MS were randomized to either an experimental (*n* = 18) or a control (*n* = 12) group. The RT group performed 10 weeks of lower limb resistance training with a concentric phase at maximum velocity. Static balance was measured before and after intervention. Results: No significant group × time interaction effects were found (ANOVA test) in any of the variables at the end of the intervention. No intragroup differences were found before or after the intervention in the balance variables. Conclusions: Resistance training with a concentric phase at maximum velocity showed no impact on balance in our sample. Future studies should examine programs of longer duration or combined with other types of training, such as balance training, with the aim of obtaining improvements in this variable in people with MS.

## 1. Introduction

Multiple sclerosis (MS) is a chronic progressive disease of the central nervous system that disrupts a wide range of functions, including cognition, muscle strength, coordination, vision, speech, and sensation [1,2,3]. Some of these symptoms cause gait and balance impairments [4]. In addition, balance is impaired in people with MS, even in the absence of clinical disability [4]. This impairment can lead to an increase in risk of falls, as well as decreased autonomy and mobility [5].

According to the available evidence, it is generally established that people with MS have worse balance control than healthy people. Analyses of balance using a force platform show greater oscillations in the lateral and sagittal planes in people with MS compared to non-MS subjects [5,6,7]. In addition, studies demonstrate that those individuals with MS who presented greater displacements of the center of pressure during the static balance test are at greater fall risk than those with smaller displacements [5,7,8]. Similarly, Sosnoff et al. [5] showed that those patients classified as ‘fallers’ presented higher displacement velocities in the mediolateral (SDV_ML_) and anteroposterior (SDV_AP_) planes as well as in the total displacement velocity (MTV) than ‘non-fallers’. From a biomechanical point of view, the displacement of the center of pressure (measured through stabilometry variables) represents a marker of energy expenditure required to maintain balance [9]. Therefore, an improvement in these variables would lower energy expenditure while standing. A better energy economy could reduce fatigue during walking or the performance of daily living tasks in people with MS. Therefore, it is important to include tools in the rehabilitation process of patients with MS that have the capacity to improve balance variables.

Improving balance is a vital goal of rehabilitators because of the close relationship between balance and the risk of falls or the need to use balance aids [5]. Therefore, in recent years, there has been a growing interest in the scientific community to assess and improve balance in this population. Multisensory training programs, aerobic training, resistance training, yoga, pilates, tai chi, and various neurotherapeutic strategies have been some of the most widely used protocols by rehabilitators, trainers, and scientists [10,11,12]. However, although some of these types of training are widely used and have shown small benefits in balance, these improvements are generally not clinically relevant. Therefore, it is necessary to modify some of the variables of these types of training to enhance improvements in balance.

Previous studies have analyzed the effects of traditional resistance training on balance, finding moderate benefits in the MS population [13,14]. However, according to a recent study, the control of movement velocity in resistance training program is a variable that could modulate the neuromuscular adaptations [15]. Therefore, resistance training with the concentric phase at maximum velocity (FVCRT) could be an alternative type of training that enhances the benefits of the neuromuscular system, and consequently improves balance in the population with MS. In this context, to the best of our knowledge, only one study has analyzed the benefits of FVCRT on balance in people with MS [16]. This study found improvements after 8 weeks of FVCRT in a sample of 7 people with moderate MS disability. However, the small sample size, the use of clinical tests and not posturography, as well as the lack of a control group in the study by Karpatkin et al. [16] requires that a randomized clinical trial (RCT) be conducted to confirm the promising results previously found. 

Although many studies have examined the benefits of exercise on balance in people with MS, there is insufficient evidence to show the real effects of resistance training programs, and specifically FVCRT, on static balance, measured by force platform, in people with MS. Therefore, the main objective of this RCT was to analyze the effects of a 10 week lower limb FVCRT on static balance with open and closed eyes in people with MS. Our hypothesis was that FVCRT would have a positive effect on balance.

## 2. Materials and Methods

### 2.1. Study Design and Testing Procedure

A 10 week randomized, controlled, single-blinded intervention was conducted in the UCAM Sport Center (Murcia, Spain). Participants were randomly assigned to either a resistance training group [RTG] (*n* = 18) or control group [CG] (*n* = 12) All testing sessions were performed at the same time of day to avoid differences in circadian rhythms, and the temperature (21–22 °C) and humidity (55–60%) were regulated. Balance was measured before and after the FVCRT in both RTG and CG. The Catholic University of Murcia’s Science Ethics Committee approved this study in accordance with the Declaration of Helsinki [17]. The consort guidelines for RCT were followed, and the study was registered at ClinicalTrials.gov (identifier: NCT04452760).

### 2.2. Participants

Thirty people with MS were recruited through the local MS association. A board-certified neurologist diagnosed the participants with relapsing–remitting or secondary progressive MS, according to the McDonald criteria [18]. A randomization table with a ratio of 3:2 (3 participants to RTG, 2 participants to CG) randomly allocated the participants to the groups. To be included in the study, people with MS had to be in the stable phase of the disease and ambulatory (walk independently for >100 m). The exclusion criteria were as follows: (1) Expanded Disability Status Scale ≤ 1 or ≥6; (2) experienced a relapse within the prior 12 months; (3) used corticosteroid treatment within the last 2 months; (4) involved in a training program within the prior 4 months. Signed informed consent was obtained prior to the start of the study. If a participant experienced an attack influencing the pyramidal functions or if they completed fewer than 90% of the planned training sessions, they were excluded from the study or final analysis. 

### 2.3. Procedures

The RTG completed 10 weeks of lower-limb FVCRT, three times per week on alternating days. Forty-eight hours of rest was provided between sessions. Participants began with a standardized warm-up that consisted of 5 min on a stationary bicycle, mobility of lower-limbs, and 5 repetitions at 40% 1-RM on each machine. Next, 4 lower-limb exercises (leg press, leg extension, hip extension, and seated calf raise) were performed on conventional weight machines (Technogym, Cesena, Italy). Unilateral leg press and hip extension were performed to account for strength differences between limbs. Previous recommendations were used for intensity (60–75% 1-RM), sets (2–4), repetitions (8–15), and rest between sets (120 s) [15,19]. See Supplementary Table S1 for more details regarding the training protocol. Participants were told to avoid muscle failure and to leave 2 repetitions in reserve. In addition, participants were instructed to lower the weight in a controlled manner with a brief pause at the end, followed by fast, maximal force production in the concentric phase to maximally engage the neural component [15]. The training load for each exercise was individualized based on the 1-repetition maximum (1-RM), which was calculated before study commencement. The 1-RM load was estimated by having the participants complete 4 sets of each exercise with the following procedure: 1 set of 10 repetitions at 50% of the perceived 1-RM, 1 set of 5 repetitions at 75% of the perceived 1-RM, and 1 set of 1 repetition at 100% of 1-RM. Five minutes of rest was given between sets. If more than 1 repetition in the last set could be performed, then the 1-RM was estimated using previous recommendations [20,21]. The load intensified by 2–5% if the participants completed 2 or more repetitions than the stipulated ones, always leaving 2 repetitions in reserve [22]. At the end of each session, an exercise diary was recorded for each participant (e.g., type of exercise, the weight lifted, the repetition number, and the number of completed sets). The same researcher, specialized in strength and conditioning training and certified by NSCA-CPT, supervised all of the exercise sessions (groups of 4 participants). No intervention was provided to the CG. 

### 2.4. Outcomes Measures

A different investigator performed the pre–post testing measurements and was blinded to group allocation. Due to the nature of the intervention (resistance training vs. no exercise), participants were not blinded to the intervention. However, CG participants were invited to perform the 10 weeks of lower-limb FVCRT after the termination of the study. Static balance was measured in eyes-closed and eyes-open conditions. The outcome measures were: the mean anterior/posterior displacement (MAPD; mm), mean medial/lateral displacement (MMLD; mm), total sway displacement (TSD; mm), sway area (SA; mm^2^), mean total velocity (MTV; mm/s), phase plane portrait anterior/posterior (PPP_AP_; a.u.), medial/lateral (PPP_ML_; a.u.) and anterior/posterior–medial/lateral (PPP_AP-ML_; a.u.), standard deviation of velocity anterior/posterior (SDV_AP_; mm/s) and medial/lateral (SDV_ML_; mm/s), and standard deviation of amplitude anterior/posterior SDA_AP_ (mm) and medial/lateral SDA_ML_ (mm).

### 2.5. Testing Procedures

Each assessment was conducted by the same researcher. Prior to testing, a standardized warm-up of 5 min on a cycle ergometer at 50 W and a dynamic stretching routine were performed. Prior to the start of the study, participants came to the laboratory to familiarize themselves with the tests, then 48 h later they returned to carry out the measurements. The same tests were carried out following the 10 week intervention.

### 2.6. Balance

During the static balance measurements, the participant stood quietly over a portable force platform (Kistler 9286BA, Kistler Group, Winterhur, Switzerland) barefoot, with the legs shoulder-width apart and the arms hanging at the sides. Each participant completed two 30 s trials with the eyes open and two 30 s trials with the eyes closed, before and after the 10 week intervention. A two minute rest period was provided between trials. The trial with the best balance results for each test was analyzed. The MAPD, MMLD, TSD, SA, MTV, PPP_AP_, PPP_ML_, PPP_AP-ML_, SDV_AP_, SDV_ML_, SDA_AP_, and SDA_ML_ variables were calculated using the following formulas [23]:

$$MAPD=\frac{\sum ABS\left(Y_{i}-\frac{\sum \left(Y_{i}\right)}{N}\right)}{N}$$

$$MMLD=\frac{\sum ABS\left(X_{i}-\frac{\sum \left(X_{i}\right)}{N}\right)}{N}$$

$$TSD=\sum \sqrt{{\left(Y_{i+1}-Y_{i}\right)}^{2\;}+{\left(X_{i+1}-X_{i}\right)}^{2}}$$

$$Area=2\pi F_{0.05\left[2N-2\right]}\sqrt{\sigma_{x}^{2}\sigma_{y}^{2}-\sigma_{xy}^{2}},\;\mathrm{where}\;\sigma_{xy}=\sum \frac{\left(x_{i}-\bar{x}\right)\left(y_{i}-\bar{y}\right)}{N}$$

$$MTV=\frac{1}{T}\sum_{1}^{T}\sqrt{{\left(x_{t+1}-x_{t}\right)}^{2}+{\left(y_{t+1}-y_{t}\right)}^{2}}$$

$$PPP_{AP}=\sqrt{\sigma_{x}^{2}+\sigma_{vx}^{2}}$$

$$PPP_{ML}=\sqrt{\sigma_{y}^{2}+\sigma_{vy}^{2}}$$

$$PPP_{AP-ML}=\sqrt{\sigma_{\tau x}^{2}+\sigma_{\tau y}^{2}}$$

$$SDV_{AP}=\sqrt{\frac{\sum {\left(v_{xi}-\bar{v}\right)}^{2}}{N-1}},\;\mathrm{where}\;v_{xi}=\frac{x_{i+1}-x_{i}}{t_{i+1}-t_{i}}$$

$$SDV_{ML}=\sqrt{\frac{\sum {\left(v_{yi}-\bar{v}\right)}^{2}}{N-1}},\;\mathrm{where}\;v_{yi}=\frac{y_{i+1}-y_{i}}{t_{i+1}-t_{i}}$$

$$SDA_{AP}=\sqrt{\frac{\sum {\left(x_{i}-\bar{x}\right)}^{2}}{N-1}}$$

$$SDA_{ML}=\sqrt{\frac{\sum {\left(y_{i}-\bar{y}\right)}^{2}}{N-1}}$$

### 2.7. Statistical Analyses

R software (3.6.0) was used to perform the statistical analyses. Values over 3 standard deviations were considered outliers. The Shapiro–Wilk test verified the normality of the group distributions, while Bartlett’s test determined the homogeneity between variances. In case of non-normality, the non-parametric Fligner test together with Levene’s test were performed. At baseline, no differences between groups were found with the Bayes factor (Bayes factors < 0.3, 0.3–1, 1–3, and >3 represents evidence of no difference, equal evidence, moderate evidence of difference, and strong evidence of difference, respectively), as well as with the *t*-test in case of normality and with the Wilcoxon test and the nonparametric permutation test in case of non-normality.

In addition, a two-way mixed ANOVA analysis (in cases of bad violation of the assumptions, mainly the homogeneity of variances assumption, the Kruskall–Wallis test would be performed as the nonparametric version of the ANOVA test, whose effects would be interpreted with the Wilcoxon test, although in our case this was not necessary) was performed for each variable to analyze the effects of the trial with a general linear model with two time points (pre and post) and two groups (RTG and CG). Together with the assumptions of normality and variance, the assumption of homogeneity in covariances was tested with Box’s M test, and the Mauchly sphericity correction was applied. Post-hoc effects were evaluated with a pairwise t-test with Bonferroni correction.

In addition, the effect size was determined via general eta squared (η) test for variance analysis and Cohen’s d to calculate the standardized difference between two means. Here, η values around 0.01, 0.06, and >0.14 represented small, medium, and large effects, respectively. Cohen’s d values of 0.2, 0.5, and 0.8 represented small, moderate, and large effects, respectively. Statistical significance was established at *p* < 0.05. Furthermore, a two-way mixed ANCOVA test was performed to analyze possible covariate controls (age, EDSS, and height).

On the other hand, for each of the trials, a multivariate test was performed to evaluate the significance of the training as a whole. In this case, possible outliers were evaluated using Mahalanobis distance and normality was evaluated with the multivariate Shapiro–Wilk test. The difference between pre and post means was analyzed with the Hotelling test, whereas the homogeneity in covariances was evaluated with the Box’s M test and a two-way RM MANOVA was performed to evaluate interactions with time and group by also controlling for covariates in two-way MANCOVA.

## 3. Results

**Figure 1:**
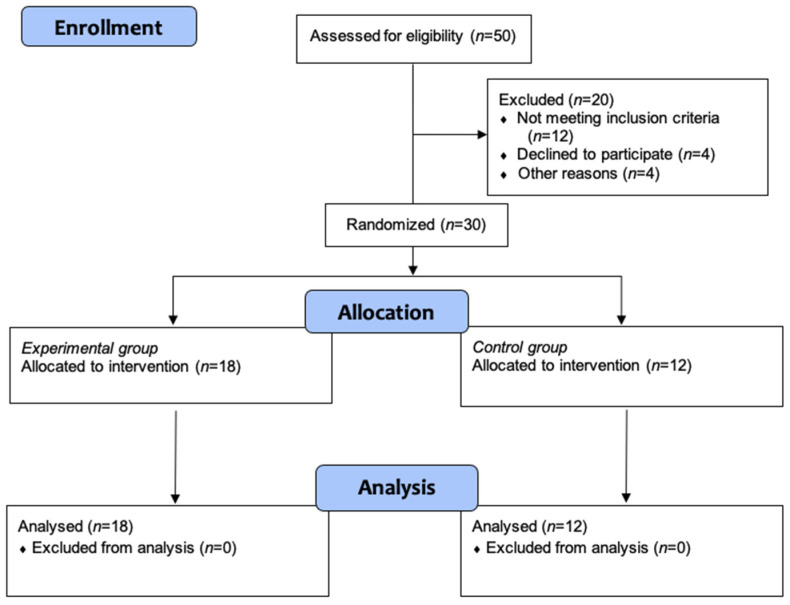
Study flowchart.

Participant’s characteristics and a flowchart are presented in Table 1 and Figure 1, respectively. All participants completed the intervention and were included in the data analysis. No participant showed adverse effects related to the resistance training program.

Firstly, in the open and closed eyes conditions, no difference was observed between groups at baseline in any of the variables (Table 2 and Table 3).

However, a moderate effect was found on PPP_AP_ in the eyes-open balance test and SDV_ML_ in eyes-closed balance test after the training. In addition, there was a trend where the RTG maintained their values, whereas it worsened in the CG. However, the variance observed in the eyes-closed measurements were high and prevented the detection of a significant effect (Table 4 and Table 5).

Finally, some marginal significant interaction effects were found through the ANOVA test, where for most of the variables the p-value of the time x group effect were between 0.05 and 0.10. It can also be noted that in most of the cases, RTG measurements tended to remain consistent, whereas the CG measurements tended to increase. The small sample size and high variance of the measurements seem to be the main drawbacks to obtaining clear significant changes (Table 6).

No significant differences were found when controlling for the covariates of age, EDSS, and height in the ANCOVA test. No significant differences were found in the multivariate test (Table 7).

## 4. Discussion

Our study aimed to analyze the efficacy of a 10 week resistance training program on static balance in people with MS. Contrary to our hypothesis, FVCRT did not have a significative impact on static balance in our sample.

The proliferation of clinical tests and scales assessing balance (i.e., Berg Balance Scale or Timed Up and Go Test) in the MS population has increased substantially [24]. However, in recent years, the use of computer-based force platform measures, for example static or dynamic posturography, has been introduced, since such tools provide a more objective and reliable analysis of balance in this population. The use of force platforms can detect problems or improvements in balance that may be undetectable by other clinical scales [25]. Due to the economic costs, most clinical studies examining the effects of exercise on balance have used the Berg Balance Scale, while only a few studies, including this study, have used a force platform. Therefore, the novelty of this study is the use of a force platform to measure static balance after a FVCRT program.

To our knowledge, only one study has analyzed the efficacy of resistance training on balance, measured via force platform, in people with MS. Similar to our results, De Bolt and McCubbin [26] found no change in balance (anteroposterior sway (MAPD), mediolateral sway (MMLD), and sway velocity (TSD)) after an 8 week period of home-based resistance training. In addition, other studies also measured the efficacy of different rehabilitation strategies, such as combined training or specific balance training, on balance measured with this instrument. The results found by these studies differ from one another. Widener et al. [27] found no improvement in balance (TSD) after a balance-based torso weightlifting intervention. However, Schuhfried et al. [28], who used a whole-body vibration training intervention, and Missaoui et al. [29], who performed a proprioceptive rehabilitation, did find improvements in balance (sway area (SA)) after the training programs.

On the other hand, we found up to 8 studies that used the Berg Balance Scale to measure balance after resistance training programs. Of these, some studies found moderate improvements [14,16,30,31,32], while others, in line with our results, found no change in balance after the intervention [33,34,35]. Of these, only Karpatkin et al. [16] performed a FVCRT (8 weeks, single exercise intervention (unilateral leg press), with two training sessions per week, 4 sets of 4 repetitions at 85–95% 1-RM). The management of variables differs among all studies, with durations ranging from 4 [33] to 12 weeks [14,30], training frequencies ranging from 2 [34] to 5 workouts per week [33], and intensity levels ranging from body weight [30] to 85–95% 1-RM [16]. This high heterogeneity in training protocols may be the cause of the lack of consensus in the results. In addition, the use of clinical tests in the aforementioned studies instead of force platform analysis may also lead to a biased view of the results.

In addition, there are some factors that may explain the absence of changes found in static balance in our study. The first of these may be training duration, since it has been observed that training programs of longer duration (>12 weeks) provide greater adaptations in balance [14,30]. Second, the selection of exercises performed in our program may be influential. The participants only executed single-joint exercises. Previous evidence shows that multi-joint exercises (squat, leg press, etc.) improve intra- and inter-muscular coordination to a greater extent than single-joint exercises [36]. Therefore, it is presumable that multi-joint exercises could have a greater impact on balance. Finally, our study consisted of an intervention with resistance training only. However, specific sensory integration balance training [37] combined with resistance training could improve balance to a greater extent in populations with motor control problems, such as people with MS.

Moreover, and following the recommendations proposed by Prosperini et al. [25], manipulating one or more specific inputs for postural control (visual, vestibular, or proprioceptive) during a balance test on force platforms may give us information on the contribution of the motor and sensory components to balance control. In our study, we manipulated the visual stimuli (eyes-open vs. eyes-closed conditions) in order to quantify the differences between the two tests after the 10 weeks of intervention. As expected, in our sample, worse values were found in the pre-intervention measurements in the eyes-closed test than in the eyes-open test in both groups. However, post-intervention changes were similar and almost imperceptible in both conditions. These results show that neither the motor nor the sensory component improved after the resistance training program. Since balance is influenced by the motor and sensory components, future studies should combine training focused on improving the motor component (such as resistance training) together with other types of training that have a greater capacity to improve the proprioceptive and sensory components.

### Limitations of the Study

The study has some limitations that should be mentioned, and consequently the results should be interpreted with caution. First, the sample was composed of people with MS with a wide range of EDSS (1–6). Therefore, the data in the balance tests, both in the open-eyed and closed-eyed conditions, had very high standard deviations, which prevented the results from having greater statistical power. The high standard deviations may be explained by the low sample size and the different sample sizes of the experimental and control groups. Second, the duration of the program was only 10 weeks long. It is recommended that longer training durations be used in future studies to assess the long-term effects of FVCRT on balance. Third, dynamic balance was not evaluated in our study. It would be interesting if in future studies dynamic balance, a variable closely related to mobility or autonomy, could also be analyzed. Finally, as a future line of research, it is recommended that larger-scale clinical trials be carried out to confirm the results found in this and other studies.

## 5. Conclusions

Resistance training developing the concentric phase at maximum velocity did not impact static balance in our sample of people with MS. Combining resistance training with other types of more specific training, such as balance training, should be studied in the future.

## Figures and Tables

**Table 1:** Participant characteristics.

Characteristics	All (*n* = 30)	RTG (*n* = 18)	CG (*n* = 12)	*p*
Age (years)	46.21 ± 10.43	44.89 ± 10.62	48.36 ± 10.23	0.394
Sex (men/women)	15:15	10:8	5:7	
MS phenotype (RR/SP)	27:3	16:2	11:1	
EDSS	3.21 (1.00–6.00)	3.17 (1.00–6.00)	3.27 (1.50–5.50)	
Weight (kg)	68.51 ± 11.55	67.19 ± 10.63	70.67 ± 13.17	0.442
Height (cm)	166.86 ± 6.95	166.44 ± 7.32	167.54 ± 6.58	0.687
BMI (kg⋅m^−2^)	24.56 ± 3.29	24.26 ± 3.12	25.06 ± 3.64	0.534
Fat mass (%)	26.47 ± 8.72	25.92 ± 8.28	27.34 ± 9.69	0.680

Data are presented as means ± SD. Significance was set at *p* = 0.05. BMI: Body Mass Index; CG: Control Group; EDSS: Expanded Disability Status Scale; MS: Multiple Sclerosis; RR: Relapsing–Remitting; RTG: Resistance Training Group; SP: Secondary-Progressive.

**Table 2:** Group comparisons of baseline measurements in eyes-open balance test.

Outcomes	All (*n* = 30)	RTG (*n* = 18)	CG (*n* = 12)	*p*
SA (mm^2^)	5.35 ± 4.11	5.00 ± 3.93	5.49 ± 3.76	0.731
TSD (mm)	917.00 ± 183.00	914.00 ± 191.00	870.00 ± 149.00	0.493
MAPD (mm)	2.24 ± 1.06	2.04 ± 1.10	2.54 ± 1.21	0.264
MMLD (mm)	4.20 ± 1.80	4.39 ± 2.00	4.15 ± 1.67	0.729
SDA_AP_ (mm)	5.23 ± 2.20	5.38 ± 2.27	5.16 ± 2.06	0.785
SDA_ML_ (mm)	2.85 ± 1.31	3.22 ± 1.46	2.64 ± 1.43	0.299
SDV_AP_ (mm/s)	22.57 ± 5.91	21.90 ± 4.17	22.40 ± 6.86	0.834
SDV_ML_ (mm/s)	26.45 ± 6.40	26.9 ± 6.84	24.30 ± 5.30	0.259
PPP_AP_ (a.u.)	23.32 ± 6.03	22.8 ± 4.40	23.10 ± 6.94	0.876
PPP_ML_ (a.u.)	26.77 ± 6.42	27.20 ± 6.83	24.60 ± 5.30	0.275
PPP_AP-ML_ (a.u.)	36.06 ± 7.30	35.80 ± 7.26	34.50 ± 6.48	0.629
MTV (mm/s)	30.59 ± 6.12	30.50 ± 6.38	29.00 ± 4.93	0.504

Data are presented as means ± SD. Significance was set at *p* = 0.05. CG: Control Group; MAPD: Mean Anterior/Posterior Displacement; MMLD: Mean Medial/Lateral Displacement; MTV: Mean Total Velocity; PPP_AP_: Phase Plane Portrait Anterior/Posterior; PPP_ML_: Phase Plane Portrait Medial/Lateral; PPP_AP-ML_: Phase Plane Portrait Anterior/Posterior-Medial/Lateral; RTG: Resistance Training Group; SA: Sway Area; SDA_AP_: Standard Deviation of Amplitude Anterior/Posterior; SDA_ML_: Standard Deviation of Amplitude Medial/Lateral; SDV_AP_: Standard Deviation of Velocity Anterior/Posterior; SDV_ML_: Standard Deviation of Velocity Medial/Lateral; TSD: Total Sway Displacement.

**Table 3:** Group comparisons of baseline measurements in eyes-closed balance test.

Outcomes	All (*n* = 30)	RTG (*n* = 18)	CG (*n* = 12)	*p*
SA (mm^2^)	6.68 ± 5.49	5.25 ± 5.08	6.34 ± 3.71	0.552
TSD (mm)	1105.00 ± 285.00	1085.00 ± 270.00	992.00 ± 85.80	0.316
MAPD (mm)	2.47 ± 1.68	2.14 ± 0.89	2.22 ± 1.68	0.893
MMLD (mm)	5.82 ± 3.05	5.16 ± 3.03	6.22 ± 2.72	0.339
SDA_AP_ (mm)	7.16 ± 3.58	6.20 ± 3.48	7.65 ± 3.26	0.270
SDA_ML_ (mm)	3.14 ± 2.07	2.80 ± 2.07	2.79 ± 1.20	0.985
SDV_AP_ (mm/s)	28.61 ± 9.21	26.70 ± 7.62	28.30 ± 7.65	0.600
SDV_ML_ (mm/s)	28.13 ± 6.27	28.10 ± 6.19	26.30 ± 4.47	0.389
PPP_AP_ (a.u.)	29.55 ± 9.49	27.70 ± 8.03	29.40 ± 7.89	0.598
PPP_ML_ (a.u.)	28.44 ± 6.27	28.30 ± 6.19	26.70 ± 4.69	0.456
PPP_AP-ML_ (a.u.)	42.47 ± 10.12	41.30 ± 9.96	41.10 ± 6.93	0.947
MTV (mm/s)	35.95 ± 8.26	35.30 ± 8.45	34.60 ± 5.53	0.811

Data are presented as means ± SD. Significance was set at *p* = 0.05. CG: Control Group; MAPD: Mean Anterior/Posterior Displacement; MMLD: Mean Medial/Lateral Displacement; MTV: Mean Total Velocity; PPP_AP_: Phase Plane Portrait Anterior/Posterior; PPP_ML_: Phase Plane Portrait Medial/Lateral; PPP_AP-ML_: Phase Plane Portrait Anterior/Posterior-Medial/Lateral; RTG: Resistance Training Group; SA: Sway Area; SDA_AP_: Standard Deviation of Amplitude Anterior/Posterior; SDA_ML_: Standard Deviation of Amplitude Medial/Lateral; SDV_AP_: Standard Deviation of Velocity Anterior/Posterior; SDV_ML_: Standard Deviation of Velocity Medial/Lateral; TSD: Total Sway Displacement.

**Table 4:** Group comparisons in eyes-open balance test after intervention.

Outcomes	RTG	CG	BF	*t* Test	*p*
SA (mm^2^)	5.12 ± 4.25	6.10 ± 4.86	0.398	−0.59	0.55
TSD (mm)	893.00 ± 204.00	1006.00 ± 163.00	0.894	−1.65	0.11
MAPD (mm)	1.99 ± 0.77	2.59 ± 1.16	0.993	−1.57	0.13
MMLD (mm)	3.94 ± 1.68	4.34 ± 1.98	0.398	−0.60	0.55
SDA_AP_ (mm)	5.54 ± 2.52	4.91 ± 2.13	0.429	−0.74	0.456
SDA_ML_ (mm)	2.50 ± 0.99	3.28 ± 1.32	1.192	−1.73	0.097
SDV_AP_ (mm/s)	21.20 ± 5.85	25.80 ± 6.81	1.377	−1.88	0.074
SDV_ML_ (mm/s)	26.00 ± 6.42	28.50 ± 6.79	0.504	−0.97	0.341
PPP_AP_ (a.u.)	22.00 ± 6.01	26.40 ± 6.84	1.278	−1.84	0.080
PPP_ML_ (a.u.)	26.30 ± 6.52	29.00 ± 6.67	0.553	−1.10	0.284
PPP_AP-ML_ (a.u.)	34.90 ± 8.01	39.70 ± 6.66	1.040	−1.78	0.087
MTV (mm/s)	29.80 ± 6.81	33.50 ± 5.49	0.861	−1.62	0.117

Data are presented as means ± SD. Significance was set at *p* = 0.05. CG: Control Group; MAPD: Mean Anterior/Posterior Displacement; MMLD: Mean Medial/Lateral Displacement; MTV: Mean Total Velocity; PPP_AP_: Phase Plane Portrait Anterior/Posterior; PPP_ML_: Phase Plane Portrait Medial/Lateral; PPP_AP-ML_: Phase Plane Portrait Anterior/Posterior-Medial/Lateral; RTG: Resistance Training Group; SA: Sway Area; SDA_AP_: Standard Deviation of Amplitude Anterior/Posterior; SDA_ML_: Standard Deviation of Amplitude Medial/Lateral; SDV_AP_: Standard Deviation of Velocity Anterior/Posterior; SDV_ML_: Standard Deviation of Velocity Medial/Lateral; TSD: Total Sway Displacement.

**Table 5:** Group comparisons in eyes-closed balance test after intervention.

Outcomes	RTG	CG	BF	*t* Test	*p*
SA (mm^2^)	6.84 ± 5.81	9.16 ± 6.85	0.506	−0.94	0.347
TSD (mm)	1123 ± 372	1134.00 ± 208.00	0.373	−0.08	0.937
MAPD (mm)	2.64 ± 1.99	2.94 ± 1.77	0.387	−0.41	0.68
MMLD (mm)	5.82 ± 3.19	6.48 ± 3.38	0.395	−0.52	0.61
SDA_AP_ (mm)	7.30 ± 3.89	8.01 ± 3.65	0.390	−0.50	0.624
SDA_ML_ (mm)	3.35 ± 2.44	3.70 ± 2.16	0.390	−0.38	0.705
SDV_AP_ (mm/s)	28.60 ± 11.1	32.10 ± 9.79	0.471	−0.83	0.406
SDV_ML_ (mm/s)	27.50 ± 6.74	31.00 ± 7.00	0.679	−1.30	0.207
PPP_AP_ (a.u.)	29.70 ± 11.4	32.70 ± 10.2	0.433	−0.68	0.496
PPP_ML_ (a.u.)	27.70 ± 6.70	31.50 ± 6.88	0.744	−1.40	0.176
PPP_AP-ML_ (a.u.)	42.00 ± 12.10	46.70 ± 9.67	0.554	−1.13	0.271
MTV (mm/s)	35.60 ± 9.66	39.10 ± 8.02	0.519	−1.02	0.317

Data are presented as mean ± SD. Significance was set at *p* = 0.05. CG: Control Group; MAPD: Mean Anterior/Posterior Displacement; MMLD: Mean Medial/Lateral Displacement; MTV: Mean Total Velocity; PPP_AP_: Phase Plane Portrait Anterior/Posterior; PPP_ML_: Phase Plane Portrait Medial/Lateral; PPP_AP-ML_: Phase Plane Portrait Anterior/Posterior-Medial/Lateral; RTG: Resistance Training Group; SA: Sway Area; SDA_AP_: Standard Deviation of Amplitude Anterior/Posterior; SDA_ML_: Standard Deviation of Amplitude Medial/Lateral; SDV_AP_: Standard Deviation of Velocity Anterior/Posterior; SDV_ML_: Standard Deviation of Velocity Medial/Lateral; TSD: Total Sway Displacement.

**Table 6:** Eyes-open balance test.

				ANOVA													
				Time Effect			Group Effect			Time × Group Effect							
Outcome	Group	Pre	Post	*F*	*p*	*eta*	*F*	*p*	*eta*	*F*	*p*	*eta*	Post Intr Padj	Post Intr ES (d)	Post M	Post Intr Pad	Post Intr ES (d)
SA (mm^2^)	CG	5.49 ± 3.76	6.10 ± 4.86	0.579	0.453	0.002	0.244	0.625	0.008	0.273	0.605	0.001	0.99	0.14	Pre	1.00	0.22
	RTG	5.00 ± 3.93	5.12 ± 4.25										1.00	0.03	Post	1.00	0.13
TSD (mm)	CG	870 ± 149	1006 ± 163	1.608	0.216	0.025	0.450	0.508	0.009	3.012	0.094	0.046	0.14	0.87	Pre	0.25	0.60
	RTG	914 ± 191	893 ± 204										1.00	0.11	Post	1.00	0.25
MAPD (mm)	CG	2.54 ± 1.21	2.59 ± 1.16	0.000	0.995	0.000	2.163	0.153	0.007	0.145	0.707	0.000	1.00	0.04	Pre	0.51	0.63
	RTG	2.04 ± 1.10	1.99 ± 0.77										1.00	0.05	Post	0.21	0.44
MMLD (mm)	CG	4.15 ± 1.67	4.34 ± 1.98	0.334	0.568	0.001	0.015	0.903	0.000	2.084	0.160	0.008	0.96	0.10	Pre	1.00	0.22
	RTG	4.39 ± 2.00	3.94 ± 1.68										0.35	0.24	Post	1.00	0.13
SDA_AP_ (mm)	CG	5.16 ± 2.06	5.54 ± 2.52	0.035	0.853	0.001	0.066	0.799	0.002	3.004	0.094	0.009	0.31	0.19	Pre	0.54	0.17
	RTG	5.38 ± 2.27	4.91 ± 2.13										0.16	0.21	Pos	0.67	0.24
SDA_ML_ (mm)	CG	2.64 ± 1.43	3.28 ± 1.32	0.068	0.797	0.000	2.156	0.154	0.067	0.433	0.516	0.002	0.77	0.04	Pre	0.30	0.69
	RTG	3.22 ± 1.46	2.50 ± 0.99										1.00	0.12	Post	0.08	0.40
SDV_AP_ (mm/s)	CG	22.40 ± 6.86	25.80 ± 6.81	1.704	0.202	0.013	1.692	0.204	0.045	3.898	0.058	0.030	0.05	0.49	Pre	0.82	0.72
	RTG	21.90 ± 4.17	21.20 ± 5.85										1.00	0.13	Post	0.06	0.09
SDV_ML_ (mm/s)	CG	24.30 ± 5.30	28.50 ± 6.79	1.291	0.266	0.016	0.002	0.966	0.000	3.217	0.084	0.039	0.17	0.68	Pre	0.56	0.37
	RTG	26.90 ± 6.84	26.00 ± 6.42										1.00	0.14	Post	0.67	0.42
PPP_AP_ (a.u.)	CG	23.10 ± 6.94	26.40 ± 6.84	1.557	0.222	0.011	1.475	0.235	0.040	4.23	0.049	0.030	0.04	0.48	Pre	1.00	0.70
	RTG	22.8 ± 4.40	22.00 ± 6.01										1.00	0.15	Post	0.14	0.06
PPP_ML_ (a.u.)	CG	24.60 ± 5.30	29.00 ± 6.67	1.574	0.220	0.019	0.003	0.957	0.000	3.528	0.071	0.004	0.14	0.73	Pre	0.59	0.42
	RTG	27.20 ± 6.83	26.30 ± 6.52										1.00	0.13	Post	0.56	0.40
PPP_AP-ML_ (a.u.)	CG	34.50 ± 6.48	39.70 ± 6.66	1.725	0.200	0.023	0.685	0.415	0.016	3.482	0.073	0.045	0.12	0.79	Pre	1.00	0.65
	RTG	35.80 ± 7.26	34.90 ± 8.01										1.00	0.12	Post	0.19	0.18
MTV (mm/s)	CG	29.00 ± 4.93	33.50 ± 5.49	1.520	0.228	0.024	0.438	0.514	0.009	2.893	0.100	0.044	0.15	0.85	Pre	1.00	0.59
	RTG	30.50 ± 6.38	29.80 ± 6.81										1.00	0.11	Post	0.26	0.24

Data are presented as means ± SD. Significance was set at *p* = 0.05. CG: Control Group; MAPD: Mean Anterior/Posterior Displacement; MMLD: Mean Medial/Lateral Displacement; MTV: Mean Total Velocity; PPP_AP_: Phase Plane Portrait Anterior/Posterior; PPP_ML_: Phase Plane Portrait Medial/Lateral; PPP_AP-ML_: Phase Plane Portrait Anterior/Posterior-Medial/Lateral; RTG: Resistance Training Group; SA: Sway Area; SDA_AP_: Standard Deviation of Amplitude Anterior/Posterior; SDA_ML_: Standard Deviation of Amplitude Medial/Lateral; SDV_AP_: Standard Deviation of Velocity Anterior/Posterior; SDV_ML_: Standard Deviation of Velocity Medial/Lateral; TSD: Total Sway Displacement.

**Table 7:** Eyes-closed balance test.

				ANOVA													
				Time Effect			Group Effect			Time × Group Effect							
Outcome	Group	Pre	Post	*F*	*p*	*eta*	*F*	*p*	*eta*	*F*	*p*	*eta*	Post Intr Padj	Post Intr ES (d)	Post M	Post Intr Pad	Post Intr ES (d)
SA (mm^2^)	CG	6.34 ± 3.71	9.16 ± 6.85	7.106	0.013	0.039	0.710	0.407	0.024	0.559	0.462	0.003	0.10	0.51	Pre	1.00	0.23
	RTG	5.25 ± 5.08	6.84 ± 5.81										0.29	0.29	Post	0.71	0.38
TSD (mm)	CG	992 ± 85.8	1134 ± 208	3.118	0.090	0.024	0.161	0.691	0.005	1.034	0.319	0.008	0.25	0.89	Pre	0.65	0.41
	RTG	1085 ± 270	1123 ± 372										1.00	0.12	Post	1.00	0.03
MAPD (mm)	CG	2.22 ± 1.68	2.94 ± 1.77	6.786	0.015	0.031	0.033	0.857	0.001	0.662	0.423	0.003	0.15	0.57	Pre	1.00	0.05
	RTG	2.14 ± 0.89	2.64 ± 1.99										0.28	0.23	Post	1.00	0.16
MMLD (mm)	CG	6.22 ± 2.72	4.34 ± 1.98	1.559	0.223	0.006	0.582	0.452	0.019	0.304	0.586	0.001	0.95	0.08	Pre	0.70	0.36
	RTG	5.16 ± 3.03	3.94 ± 1.68										0.46	0.21	Post	1.00	0.20
SDV_ML_ (mm/s)	CG	26.30 ± 4.47	31.00 ± 7.00	2.669	0.115	0.027	0.167	0.687	0.005	4.524	0.043	0.045	0.17	0.80	Pre	0.83	0.51
	RTG	28.10 ± 6.19	27.50 ± 6.74										1.00	0.10	Post	0.40	0.32
PPP_AP_ (a.u.)	CG	29.40 ± 7.89	32.70 ± 10.2	5.823	0.023	0.019	0.402	0.532	0.015	0.316	0.579	0.001	0.02	0.37	Pre	1.00	0.21
	RTG	27.70 ± 8.03	29.70 ± 11.4										0.42	0.21	Post	1.00	0.27
PPP_ML_ (a.u.)	CG	26.70 ± 4.69	31.50 ± 6.88	2.961	0.098	0.030	0.261	0.614	0.008	4.559	0.043	0.045	0.16	0.82	Pre	0.96	0.28
	RTG	28.30 ± 6.19	27.70 ± 6.70										1.00	0.08	Post	0.34	0.55
PPP_AP-ML_ (a.u.)	CG	41.10 ± 6.93	46.70 ± 9.67	3.120	0.090	0.024	0.390	0.538	0.012	1.961	0.174	0.015	0.17	0.67	Pre	1.00	0.02
	RTG	41.30 ± 9.96	42.00 ± 12.1										1.00	0.06	Post	0.60	0.42
MTV (mm/s)	CG	34.60 ± 5.53	39.10 ± 8.02	2.490	0.127	0.020	0.227	0.638	0.007	1.988	0.171	0.016	0.21	0.65	Pre	1.00	0.09
	RTG	35.30 ± 8.45	35.60 ± 9.66										1.00	0.03	Post	0.68	0.39

Data are presented as means ± SD. Significance was set at *p* = 0.05. CG: Control Group; MAPD: Mean Anterior/Posterior Displacement; MMLD: Mean Medial/Lateral Displacement; MTV: Mean Total Velocity; PPP_AP_: Phase Plane Portrait Anterior/Posterior; PPP_ML_: Phase Plane Portrait Medial/Lateral; PPP_AP-ML_: Phase Plane Portrait Anterior/Posterior-Medial/Lateral; RTG: Resistance Training Group; SA: Sway Area; SDA_AP_: Standard Deviation of Amplitude Anterior/Posterior; SDA_ML_: Standard Deviation of Amplitude Medial/Lateral; SDV_AP_: Standard Deviation of Velocity Anterior/Posterior; SDV_ML_: Standard Deviation of Velocity Medial/Lateral; TSD: Total Sway Displacement.

## Data Availability

The data that support the findings of this study are available from the corresponding author upon reasonable request.

## Supplementary Materials

The following supporting information can be downloaded at: https://www.mdpi.com/article/10.3390/jcm11092405/s1: Table S1: Training program characteristics. File S1: Three channels of force-platform output-vertical force (FZ), moment about the medial/lateral axis (MY), and moment about the anterior/posterior axis (MX)-were sampled at 10 Hz each and used to calculate the coordinates of the instantaneous COP.
