# Supplementary material for: The Impact of Resistance Training Program on Static Balance in Multiple Sclerosis Population: A Randomized Controlled Trial Study
Source: J Clin Med. 2022 Apr 25;11(9):2405. doi: 10.3390/jcm11092405 (PMC9106059; doi:10.3390/jcm11092405)
Supplement: Supplementary file 1 [file jcm-11-02405-s001.zip › jcm-1673006-supplementary.pdf]

Supplementary Table S1. Training program characteristics.

**Week**	1	2	3	4	5	6	7	8	9	10
Intensity (% 1-RM)	60	65	70	75	60	65	70	75	75	60
Sets for exercise	2	3	3	4	2	3	3	3	4	2
Repetitions	15	13	9	8	15	13	9	8	8	15
Rest between set (s)	120	120	120	120	120	120	120	120	120	120

1-RM: one repetition máximum.

Supplementary Information File S1

Three channels of force-platform output-vertical force (FZ), moment about the medial/lateral axis (MY), and moment about the anterior/posterior axis (MX)-were sampled at 10 Hz each and used to calculate the coordinates of the instantaneous COP.

The variables used in the formulas are explained below:

- i = {0.01,0.02,…,29.99} refering to registered times.
- Xi and Yi make reference to the two-dimension spatial coordinates of the center-of-pressure (COP).
- ABS refers to absolute value.
- σx and σy refers to the standard deviation of each coordinate in the time sample.
- σxy makes reference to the covariance of both.
- vx and vy refers to the velocity (rate of change of the position) in each spatial axis.
- σvx and σvy refer to the standard deviation of each coordinate velocity.
- F refers to the Fisher-Snedecor distribution.
